# Coastal livelihood transitions under globalization with implications for trans-ecosystem interactions

**DOI:** 10.1371/journal.pone.0186683

**Published:** 2017-10-27

**Authors:** Daniel B. Kramer, Kara Stevens, Nicholas E. Williams, Seeta A. Sistla, Adam B. Roddy, Gerald R. Urquhart

**Affiliations:** 1 James Madison College and Department of Fisheries and Wildlife, Michigan State University, East Lansing, MI, United States of America; 2 National Oceanic and Atmospheric Administration, 1315 East West Highway, Silver Spring, MD, United States of America; 3 Environmental Studies Program, University of Colorado, Boulder, CO, United States of America; 4 Hampshire College, Amherst, MA, United States of America; 5 Yale School of Forestry and Environmental Studies, Yale University, New Haven, CT, United States of America; 6 Lyman Briggs College and Department of Fisheries and Wildlife, Michigan State University, East Lansing, MI, United States of America; University of Waikato, NEW ZEALAND

## Abstract

Anthropogenic threats to natural systems can be exacerbated due to connectivity between marine, freshwater, and terrestrial ecosystems, complicating the already daunting task of governance across the land-sea interface. Globalization, including new access to markets, can change social-ecological, land-sea linkages via livelihood responses and adaptations by local people. As a first step in understanding these trans-ecosystem effects, we examined exit and entry decisions of artisanal fishers and smallholder farmers on the rapidly globalizing Caribbean coast of Nicaragua. We found that exit and entry decisions demonstrated clear temporal and spatial patterns and that these decisions differed by livelihood. In addition to household characteristics, livelihood exit and entry decisions were strongly affected by new access to regional and global markets. The natural resource implications of these livelihood decisions are potentially profound as they provide novel linkages and spatially-explicit feedbacks between terrestrial and marine ecosystems. Our findings support the need for more scientific inquiry in understanding trans-ecosystem tradeoffs due to linked-livelihood transitions as well as the need for a trans-ecosystem approach to natural resource management and development policy in rapidly changing coastal regions.

## Introduction

Conservation planning and governance are often restricted to single ecological systems [1] due to governance and technical constraints [2]. Recognizing that a failure to identify and understand the ecological and socioeconomic linkages transcending ecological boundaries undermines efforts to manage threats to terrestrial, marine, and freshwater systems, recently proposed theoretical frameworks have advanced similar approaches for bridging this gap including integrated cross-realm planning [3], land-sea conservation planning [4], and integrated land-sea management [5]. Cross-ecosystem linkages include natural flows, anthropogenic threats, and socio-economic interactions [1]. This study provides an uncommon empirical example of a socio-economic interaction with the potential to modify land-sea linkages. Specifically, our study examines the spatially-explicit livelihood decisions of smallholder farmers and artisanal fishermen in response to increasing market access on the rapidly globalizing Caribbean coast of Nicaragua.

The planet’s ecosystems are under great pressure from humanity’s expanding trade, transportation, migration, and technology networks [6], factors which can create tight couplings between human and natural systems across space and time [7]. Remote and less disturbed regions of the world are quickly becoming more integrated with the global economy which may profoundly affect local ecosystems and their often natural resource-dependent, local economies [8]. Livelihood changes act as local adaptation and mitigation strategies to globalization [9, 10] including market changes [11], technology introductions [12], and migration [13] but also to climate change [14], extreme weather [15], economic crises [16], and policy shifts [17]. Smallholder agriculture and artisanal fishing are two livelihoods which are experiencing changes due to external disturbances across the globe [18, 19]. These changes have profound implications for ecosystem health, food security, and human wellbeing.

A third of the earth’s population are smallholder farmers [20]. Worldwide, farms smaller than one hectare account for 72% of all farms but only 8% of all agricultural land [21]. Smallholder farmers produce over 80% of food consumed in much of the developing world [20], and roughly three quarters of poor people in developing countries depend on subsistence agriculture for their livelihoods [22]. Small-scale fisheries are also important to livelihoods in developing countries, employing 90% of the world’s capture fishers, providing the primary animal protein source for 17% of the world’s population [23], and important in 93% of the world’s exclusive economic zones [24]. Over 40% of the world’s population reside in coastal areas amid highly productive river deltas, mangrove forests, coral reefs, and estuaries [25] linked to adjoining terrestrial systems. Many coastal residents in these ecologically heterogeneous areas adopt mixed natural resource-based livelihoods including smallholder agriculture and artisanal fishing [26, 27] Thus, the effects of globalization on livelihoods and local natural resources may be more complicated at the land-sea interface than in more ecologically homogenous regions [28]. Yet, little is known about how and why livelihoods change in dynamic, resource-rich, coastal regions as a result of major external disruptors like climate change and globalization and in particular how socio-economic changes may modify land-sea flows and processes.

To address these knowledge gaps, we examined both how and why artisanal fishing and smallholder farming are changing on the rapidly globalizing Caribbean coast of Nicaragua. Specifically, we analyzed both exit and entry decisions of fishers and farmers over space and time. We hypothesized that: 1) temporal and spatial variability in household livelihood decisions are due to different exposure to external drivers, primarily new market access; 2) exit and entry decisions are related to households’ participation in other natural-resource based livelihoods–fishing, farming, and forest product gathering; and 3) variability in household responses can be attributed to both community- and household-level characteristics. We end by discussing the implications of our results for conservation and governance of the land-sea interface.

### Study site

**Fig 1:**
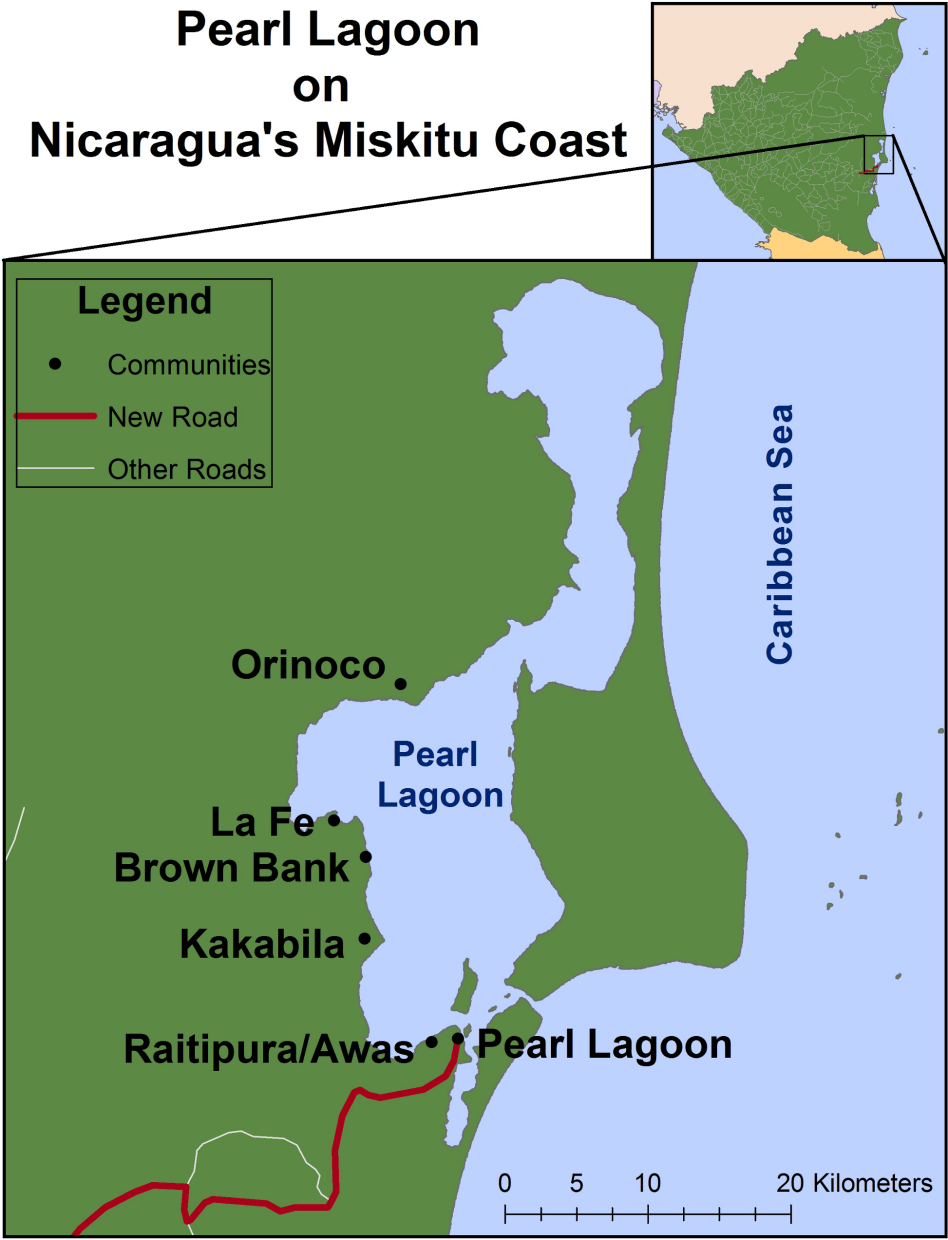
Study communities. Seven study communities including three communities (Pearl Lagoon, Awas, Raitipura) near and four communities (Kakabila, Brown Bank, La Fe, and Orinoco) far from the road completed in 2008.

Our study site included seven coastal communities located within the Región Autónoma de la Costa Caribe Sur (RACCS), an area of 27,000 km^2^ comprising more than 20% of the land area but less than 7% of the population of Nicaragua (Fig 1). Located within the Mesoamerica Biodiversity Hotspot, these small, coastal communities include people of four ethnicities: the indigenous Miskito; the Garifuna of Honduran and Caribbean origins; the Nicaraguan Kriol; and an increasing Mestizo population. Collectively, they are known as Costeños.

Historically, the RACCS has been politically and economically isolated from Pacific Nicaragua including its predominantly Mestizo culture and economic hub and capital city, Managua. For decades, Mestizos have migrated seeking land for farms and cattle and other economic opportunities. Prior to some education initiatives in the 1980s, the national government’s presence on the coast was minimal. Between the 1950s and 1970s, Mestizos, encouraged by government programs incentivizing settlement in the central highlands of Nicaragua, came to the coast intermittently, pushing an agricultural frontier from west to east, threatening the communally held lands of the RACCS [29]. However, the Caribbean coast was, and in many places remains, a remote region with minimal development [30] and only periodic interactions with other regional and global economies.

The movement of people and goods between the Nicaragua coasts increased with the completion of a transnational road in 2007. With oversight by Nicaragua’s Institute for Rural Development and funding from Japanese-financed aid program meant to assist underprivileged farmers in developing countries, the new road linked the interior, river port town of El Rama, and thereby Managua, to the coastal village of Pearl Lagoon [31]. The road’s influence on the coast’s communities, however, has not been uniform. There is no road network in the RACCS extending beyond the town of Pearl Lagoon. Therefore, residents of communities outside of Pearl Lagoon and the smaller, neighboring villages of Raitipura and Awas must travel by boat to access the new road, potentially mitigating its effects on distant communities and residents' primary livelihoods: subsistence agriculture and small-scale fishing.

Local farmers practice swidden agroforestry, some cattle ranching, and home gardening, cultivating a variety of crops primarily for subsistence purposes. This includes annual crops, perennial fruit trees, fruit-bearing herbaceous plants, and starchy roots. Working alone with occasional help from family members for the planting and harvesting of labor-intensive crops, farmers, usually men, clear land in the dry season (January-May), tend to their farms during the wet season (June-December) and harvest throughout the year. Land tenure in the region is shaped by the region’s political autonomy granted under Nicaragua’s constitution, which, theoretically, affords communal decision-making over natural resources. Community leaders sanction individual farmers’ rights to communal parcels based on indigenous and afro-descendent ethnic affiliations. In practice, these sanctions are often influenced by interpersonal relationships [32]. While selling land is not permitted, farmers can sell their rights to farm the land based on its current use and various improvements. Therefore, individuals who exit farming, if their access rights remain, can later resume farming their parcel or gain access to new land. Land for farming, generally young, phosphorus-limited, and acidic throughout the Pearl Lagoon basin [33], is readily available although new entrants may need to travel farther from community centers to access their plots. The new road has had no obvious impact on agricultural exports [31], but has led to increasing imports of fruits and vegetables from markets in the Pacific and Highland Regions of Nicaragua, subjecting local farmers to greater competition for the crops they sell.

The new road has had a much different effect on the local small-scale fishery. The 52,000 hectare Pearl Lagoon estuary, connected to the open sea and part of a larger mosaic of lagoons and rivers, supports a variety of resident and migratory fish. Fishing seasons are delineated by wet and dry periods. During the dry season, salt-water tolerant fish and shrimp return to the lagoon and freshwater species return to the rivers. Coastal fishers are artisanal, utilizing dugout canoes and fiberglass boats powered by small, outboard motors and traditional gears such as hook and line, trawl nets, gill-nets, cast-nets, and traps for lobster and crab [12]. During the Contra War of the 1980s, fish stocks saw some relief from heavy harvest pressure [30], but from the early 1990s to the present, relative biomass, mean trophic level, landings, and catch per unit effort have decreased just as average gear size, fishing transport capacity, the number of boat motors, and the number of fish buyers have increased [12].

## Methods

### Household surveys

Household surveys were completed in July of 2010 and 2012. Approximately 540 households were sampled in seven communities around the lagoon. In communities with more than 100 residents (Pearl Lagoon, Raitipura-Awas, Orinoco), we randomly sampled households while completing full censuses in the other communities. Trained students from a local university conducted face-to-face, 90–120 minute interviews covering household demographics, income and budget, material wealth, travel and communication habits, migration, social networks and relations, and livelihoods including detailed information on hunting, forest product gathering, fishing, and farming.

Between the years 2010 and 2012, local, trained, research assistants collected trip and catch data for roughly 4,000 fishing trips by fishermen in six of seven of our study communities. Data collected included dates and locations of trips, fishing gear, fishing partners, and catch details including number, weight, proportion sold to market, and price received for each species.

### Multimodel inference

We analyzed exit and entry decisions for farming and fishing households between the years 2010 and 2012 using logistic regression. For example, of the households not farming in July 2010 (i.e. first household survey), we modeled their entry decision after July 2010 but prior to July 2012 (i.e. second household survey). Similarly, of the households engaged in farming in 2010, we modeled their subsequent exit decisions prior to July 2012. While prior research has examined entry and exit decisions of commercial fishers, little is known about these decisions among artisanal fishers, and what we do know is based on fishers’ responses to hypothetical scenarios. These studies found that exit decisions were correlated with catch declines, greater material wealth, lower levels of infrastructure and economic vitality, greater livelihood diversity, lower catch values, and various demographic factors including age and educational attainment [34, 35, 36]. Even less is known about livelihood changes among smallholder farmers in the developing world [22] but similar to analyses of fishers, researchers often adopt a sustainable livelihoods approach [37] to understand these changes. Thus, households’ decisions to enter or exit farming likely depend on human capital (e.g. household size, age of household head, educational attainment of household head), natural capital (e.g. land ownership, land tenure), social capital (e.g. ethnicity, civic engagement), financial capital (e.g. household wealth), and physical capital (e.g. community infrastructure, market access). We consider these and other household- and community-level characteristic in our analyses of farm and fish exit and entry decisions on Nicaragua’s Caribbean coast.

To test our primary hypothesis that market access drives exit and entry decisions, we included a binary covariate (*Community*) for whether the household resided in one of three communities near the newly completed road (i.e. Pearl Lagoon, Awas, or Raitipura) or in one of the four distant communities (i.e. Kakabila, La Fe, Brown Bank, and Orinoco). The communities near the terminus of the new road are generally more developed and have greater access to markets via the road than communities farther away. In addition, as opposed to those nearer, communities far from the new road rely heavily on boat transportation to access markets and road vendors. Testing our hypothesis that engagement in other natural-resource based livelihoods drive exit and entry decisions, we included covariates for households’ ranking of the importance of *Fishing* and *Farming* in their livelihood portfolio and a binary covariate for engagement in forest product gathering (*Forest*). Finally, we consider various household characteristics including the head of household’s educational attainment (*Education)* and *Age*, the proportion of food self-provided by the household as a measure of *Food Self-Sufficiency*, the total number of household members (*Household Size*), and *Civic Engagement* as measured by the extent of their participation in various social activities (e.g. food and labor sharing, money lending, childcare, voting etc.). We include a nominal covariate for *Ethnicity* as determined by the head of household’s mother tongue based on Jamieson's work in the region suggesting that Miskito people were less driven by individual-oriented activities (e.g. market activities) than the Creole. While this variable is able to distinguish between Mestizo, Creole, and Miskito peoples, it cannot distinguish between the Garifuna and Creole, both of whom mainly speak Creole. Using principle component analysis, we developed a material wealth index based on the household’s possession of various goods and considered the change in wealth between 2010 and 2012 (*Household Wealth*). We included a binary variable for *Migration* which indicates whether any household member migrated outside the community in that year. Finally, modeling exit decisions, we included measures of *Catch Diversity*, the number of different marine products caught*; Crop Diversity*, the number of different crops grown; and *Farm Tenure*, the number of years since the household first farmed their primary plot (Table 1). In preliminary bivariate analyses, we tested for differences in other household capitals between entrants and non-entrants as well as between those exiting and not exiting each livelihood. Finding no significant differences, we subsequently eliminated farm area, fishing gear (i.e. crab traps, gill nets, and cast nets), livelihood diversity, and remittances from further analysis.

**Table 1:** Summary statistics for model covariates.

Variable	Description	Min	Max	Mean	Std. Dev.
Age	Age of household head	18	97	49.59	13.84
Catch Diversity	Number of fisheries in which fisher participated in 2010	0	7	3.55	1.5
Civic Engagement	Number of civic activities in which household head participated in 2012	0	10	5.45	2.55
Crop Diversity	Number of crops grown on farm in 2010	0	15	2.98	4.17
Education	Educational level of household head from 1 (none) to 10 (university degree)	1	10	3.71	2.02
Ethnicity	Nominal variable for mother tongue of household: Creole (340), Miskito (161), Spanish (19), Other (11)				
Farm Begin	Year in which began farming on primary farm plot	1901	2009	1992.61	20.67
Farming	Rank of farming in household livelihood portfolio from 1 (highest) to 6 (lowest)	1	6	4.64	1.97
Fishing	Rank of fishing in household livelihood portfolio from 1 (highest) to 6 (lowest)	1	6	3.39	2.37
Food Self-Sufficiency	Percentage of unpurchased food obtained by household in 2010	0	100	22.88	28.83
Forest	Binary variable indicating whether household participates in forest product collection (1) or not (2) in 2012	1	2	1.65	0.48
Household Size	Number of members of household	1	15	6.03	2.99
Household Wealth	Change in wealth 2010–12 based on material wealth index based on household goods possession	-7.64	5.84	0.01	1.69
Immigration	Binary variable indicating whether any household member has migrated outside community in the past year from 0 (no) to 1 (yes)	0	1	0.27	0.45

Covariates used in farming and fishing exit and entry logistic models.

We adopted an information theoretic approach to model selection [38] by constructing all possible models for each of the four response variables using the covariates noted above. Using Akaike Information Criteria for small samples (AICc), we calculated model weights across each model set. We then identified the 95% confidence set of models, which is the smallest number of models whose cumulative weights summed to 0.95. Finally, we calculated model averages for parameter estimates, their unconditional standard errors, and 95% confidence intervals across the 95% confidence set. The relative importance (RI) for individual parameter estimates was identified by summing the Akaike weights for each model that contained the parameter of interest. All statistical analyses were done in R [39] using packages ggplot2 [40], MuMIn [41], and fmsb [42].

### Ethics statement

Because our research involved human subjects, Michigan State University’s institutional review board reviewed and approved our research prior to the start of research (#07–504). Literacy rates are low in our study communities, and therefore with the approval of Michigan State University’s IRB, verbal consent was obtained from all participants before conducting household surveys. While obtaining verbal consent, the purpose and procedures of the research were explained as well as potential benefits, risks, and alternatives to participation for respondents. If verbal consent was provided, survey enumerators indicated so on a consent form before initiating the survey.

## Results

**Fig 2:**
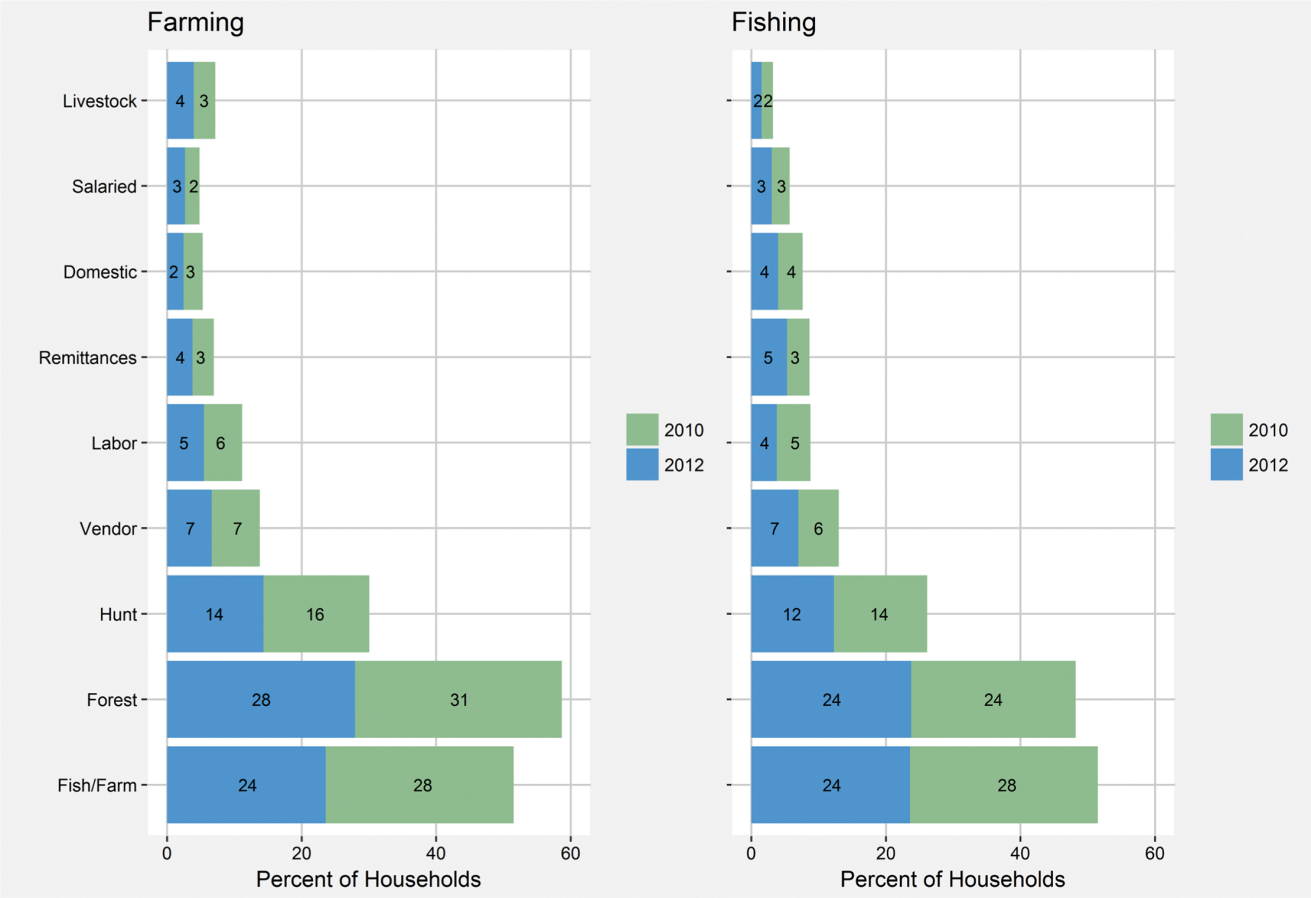
Livelihood pairings. The percent of households utilizing various livelihoods paired with farming and fishing in 2010 and 2012. Farming, fishing, and forest product gathering were the most commonly paired livelihoods.

**Fig 3:**
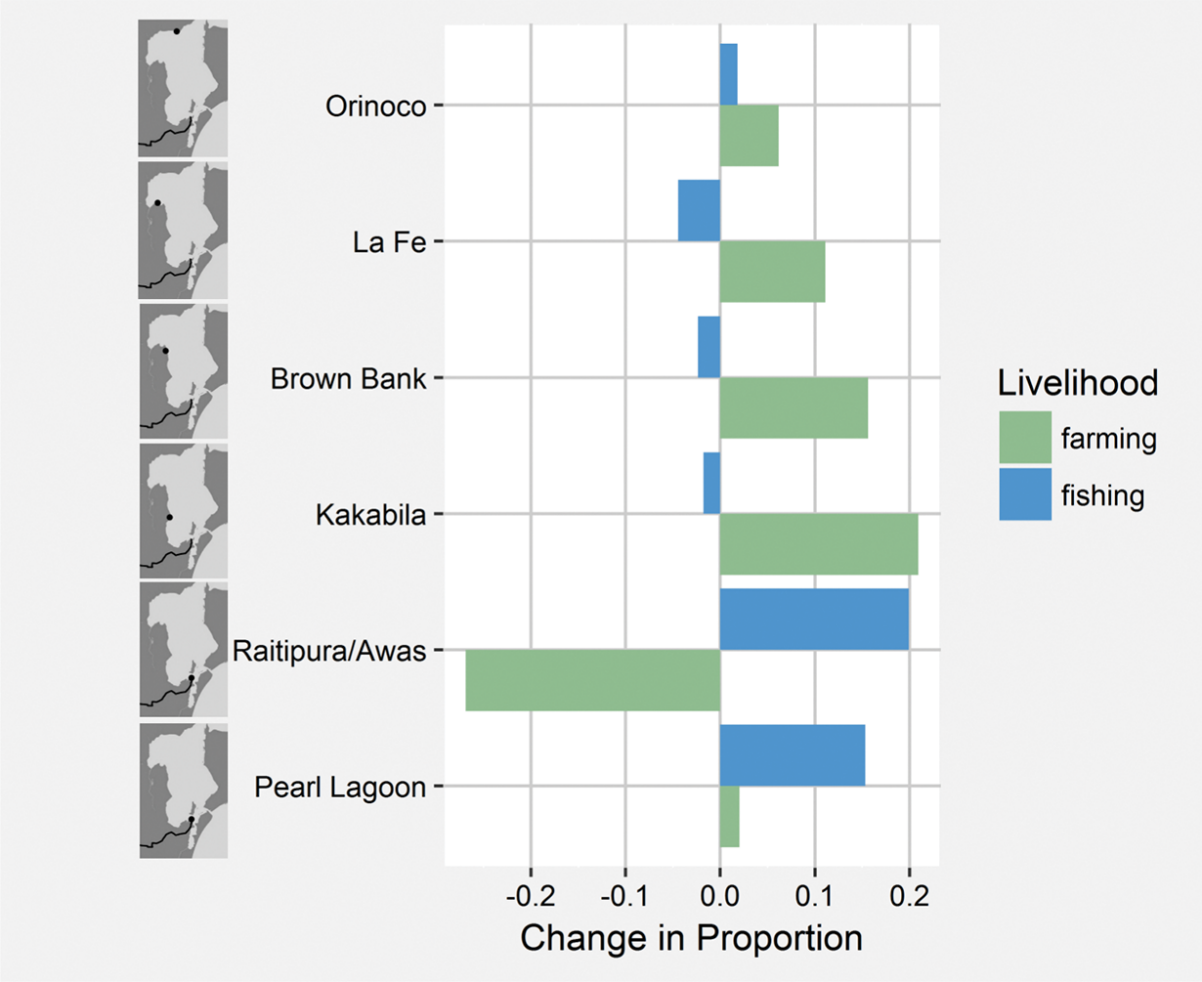
Change in fishing and farming livelihoods between 2010 and 2012. The change in the proportion of households engaging in farming and fishing livelihoods between 2010 and 2012 in seven study communities by increasing distance (bottom to top) to the newly completed road to Pearl Lagoon. The communities of Awas and Raitipura were treated as one community above due to their close proximity and shared kin networks. The figure includes only households participating in both 2010 and 2012 household surveys.

Farming, fishing, and forest product gathering were the most commonly paired livelihoods in our study communities with between one-fourth and one-third of all households participating in both fishing and farming, fishing and forest gathering, and farming and forest gathering (Fig 2). There was a strong spatial aspect to livelihood transitions over time (Fig 3). In the communities nearest the new road, the proportion of fishing households increased between 2010 and 2012. In these same communities, participation in farming decreased with the exception of a small increase in farming in Pearl Lagoon. In communities farther from the road, participation in farming increased in all four communities while fishing decreased in three of four (Fig 3). Multi-model averaging provided additional insights on these exit and entry decisions.

**Fig 4:**
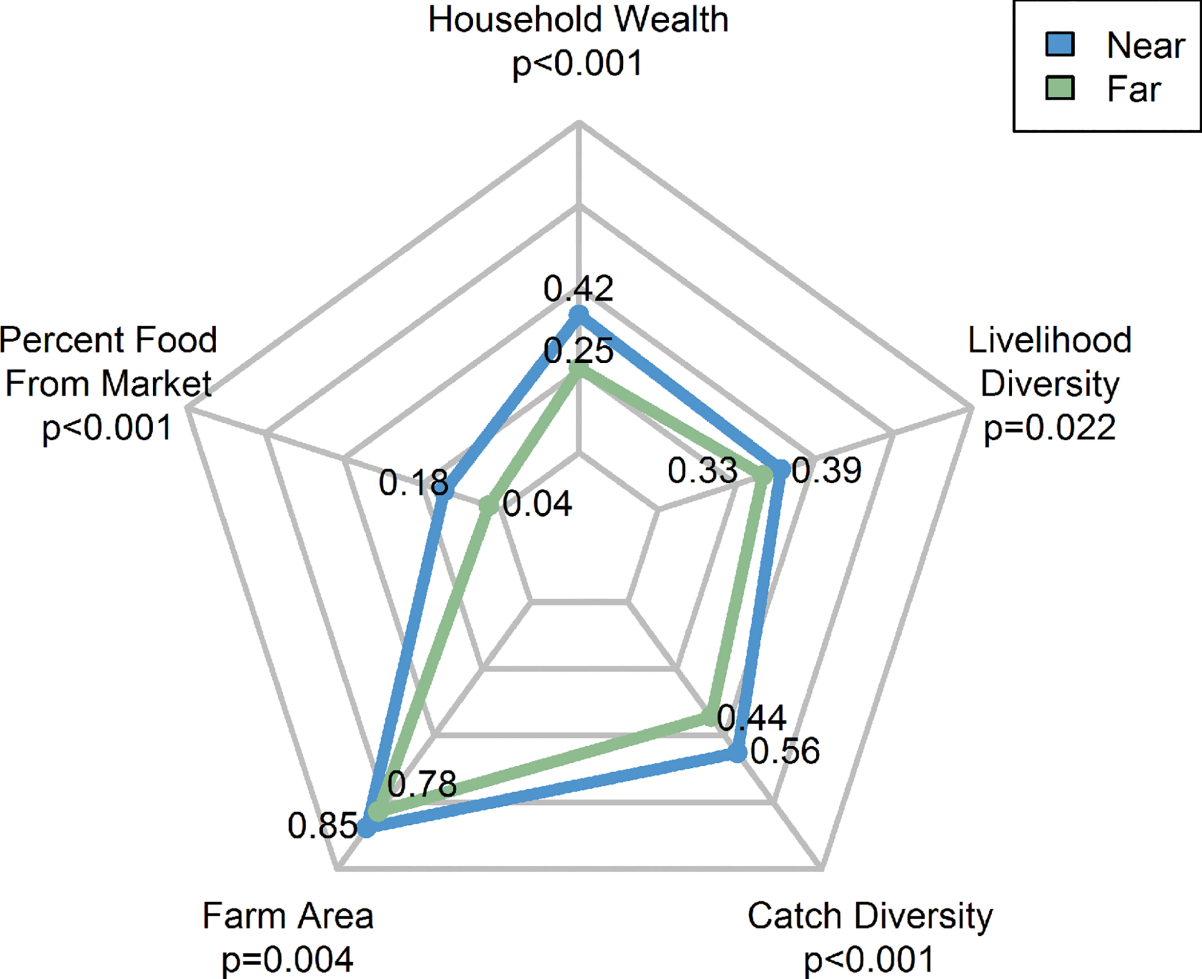
Differences between communities near and far from the new road. A multivariate comparison of the communities near and distant to the newly constructed road. Each axis depicts the normalized range (minimum to maximum) of each variable between 0 and 1. Farm area was log transformed and then normalized. The means for each variable were compared for near and distant communities using independent t-tests. See Table 1 for variable descriptions.

Whether a household was in one of the communities nearest the road versus the four distant communities was a relatively more important predictor of exiting fishing (RI = 0.95) and farming (RI = 0.91) than of entry (Table 2). Converting from log odds coefficients to odds ratios (OR), the odds of exiting farming were 387% greater for those living in communities near rather than far from the new road. The odds of exiting fishing, on the other hand, were 84% lower in near communities. In addition to proximity to the new road, near and distant communities differed in other ways. As compared to the four distant communities, the communities near the road were wealthier, received a greater proportion of their food from markets (i.e. rather than homegrown), had larger farms, harvested a greater diversity of marine products, and engaged in more livelihoods (Fig 4).

**Table 2:** Summary of multi-model results.

** **	**Farm Enter (n = 145)**					**Farm Exit (n = 96)**				
**Explanatory**	**Parameter**	**Unconditional**	**95% CI**		**Relative**	**Parameter**	**Unconditional**	**95% CI**		**Relative**
**Variables**	**Estimate**	**SE**	**Lower**	**Upper**	**Importance**	**Estimate**	**SE**	**Lower**	**Upper**	**Importance**
(Intercept)	-0.41	1.27	-2.91	2.08		-13.77	27.61	-67.89	40.35	
Age	0.00	0.02	-0.04	0.04	0.24	0.03	0.02	-0.01	0.06	0.46
Civic Engagement	0.36	0.14	0.08	0.64	**0.97**	-0.08	0.11	-0.29	0.13	0.28
Community	-0.69	0.67	-2.01	0.63	0.37	1.35	0.55	0.27	2.44	**0.91**
Crop Diversity						-0.15	0.08	-0.31	0.00	**0.74**
Education	-0.20	0.15	-0.50	0.10	0.47	0.19	0.19	-0.17	0.56	0.36
Farm Begin						0.02	0.02	-0.02	0.05	0.40
Farm Tenure						-0.02	0.02	-0.05	0.02	0.40
Fishing	-0.33	0.14	-0.61	-0.04	**0.87**	-0.07	0.11	-0.29	0.16	0.26
Food Self-Sufficiency	-0.01	0.02	-0.05	0.03	0.27	-0.02	0.01	-0.04	0.00	**0.66**
Forest	-2.36	0.66	-3.67	-1.06	**1.00**	1.08	0.51	0.09	2.08	**0.79**
Household Size	0.05	0.10	-0.15	0.24	0.26	-0.04	0.09	-0.21	0.13	0.26
Household Wealth	-0.34	0.21	-0.75	0.08	0.57	-0.15	0.16	-0.46	0.16	0.32
										
	**Fishing Enter (n = 94)**					**Fishing Exit (n = 124)**				
**Explanatory**	**Parameter**	**Unconditional**	**95% CI**		**Relative**	**Parameter**	**Unconditional**	**95% CI**		**Relative**
**Variables**	**Estimate**	**SE**	**Lower**	**Upper**	**Importance**	**Estimate**	**SE**	**Lower**	**Upper**	**Importance**
Intercept	-0.63	1.39	-3.36	2.11		-0.91	1.57	-3.98	2.16	
Age	-0.02	0.02	-0.06	0.02	0.30	0.00	0.02	-0.04	0.04	0.24
Catch Diversity						-0.31	0.21	-0.72	0.11	0.50
Civic Engagement	0.13	0.11	-0.09	0.34	0.38	-0.30	0.13	-0.54	-0.05	**0.92**
Community	-0.31	0.64	-1.57	0.94	0.26	-1.85	0.76	-3.34	-0.36	**0.95**
Education	-0.04	0.12	-0.27	0.18	0.24	0.39	0.15	0.10	0.68	**0.96**
Farming	0.11	0.18	-0.24	0.47	0.27	0.16	0.15	-0.14	0.46	0.36
Food Self-Sufficiency	0.00	0.01	-0.03	0.02	0.22	0.00	0.01	-0.02	0.03	0.25
Forest	-1.13	0.71	-2.52	0.26	0.56	1.79	0.74	0.34	3.23	**0.95**
Household Size	0.24	0.12	0.01	0.48	**0.78**	-0.05	0.08	-0.21	0.10	0.28
Household Wealth	0.51	0.16	0.19	0.83	**1.00**	0.05	0.17	-0.28	0.38	0.25

Logistic, multi-model averages for parameters based on 95% confidence set of models.

Several household characteristics were related to entry and exit decisions. *Civic Engagement* demonstrated strong relative importance for both entering farming (RI = 0.97) and exiting fishing (RI = 0.92). Greater engagement increased the odds of entering farming (OR = 1.43) and decreased the odds of exiting fishing (OR = 0.73). Having more *Education* (RI = 0.96 & OR = 1.48) also increased the odds of exiting fishing. Covariates demonstrating strong support for predicting entry into fishing were *Household Size* (RI = 0.78 & OR = 1.28) and *Household Wealth* (RI = 1.00 & OR = 1.66), both positively associated. There was moderate support for an association between increased *Food Self-Sufficiency* and exiting farming (RI = 0.66 & OR = 0.98). Households with greater *Crop Diversity* had lower odds of exiting farming (RI = 0.74 & OR = 0.86) while there was considerably weaker support for *Catch Diversity* affecting the decision to exit fishing (RI = 0.50 & OR = 0.74). Finally, while participation in *Fishing* and *Farming* demonstrated modest or weak support for understanding exit and entry decisions of farming and fishing respectively, participation in the collection of *Forest* products was highly supported in three of four model sets. The odds of entering farming were 91% more (RI = 1.00) for households gathering forest products while the odds of exiting farming were 295% less (RI = 0.79). Similarly, the odds of exiting fishing were nearly 600% less for those gathering forest products (RI = 0.95) (Table 2).

Across the four model sets, there was no support for household *Migration*, *Ethnicity*, and the *Age* of household head influencing exit and entry decisions. There was similarly weak support for *Farm Tenure* affecting farm exit decisions.

**Fig 5:**
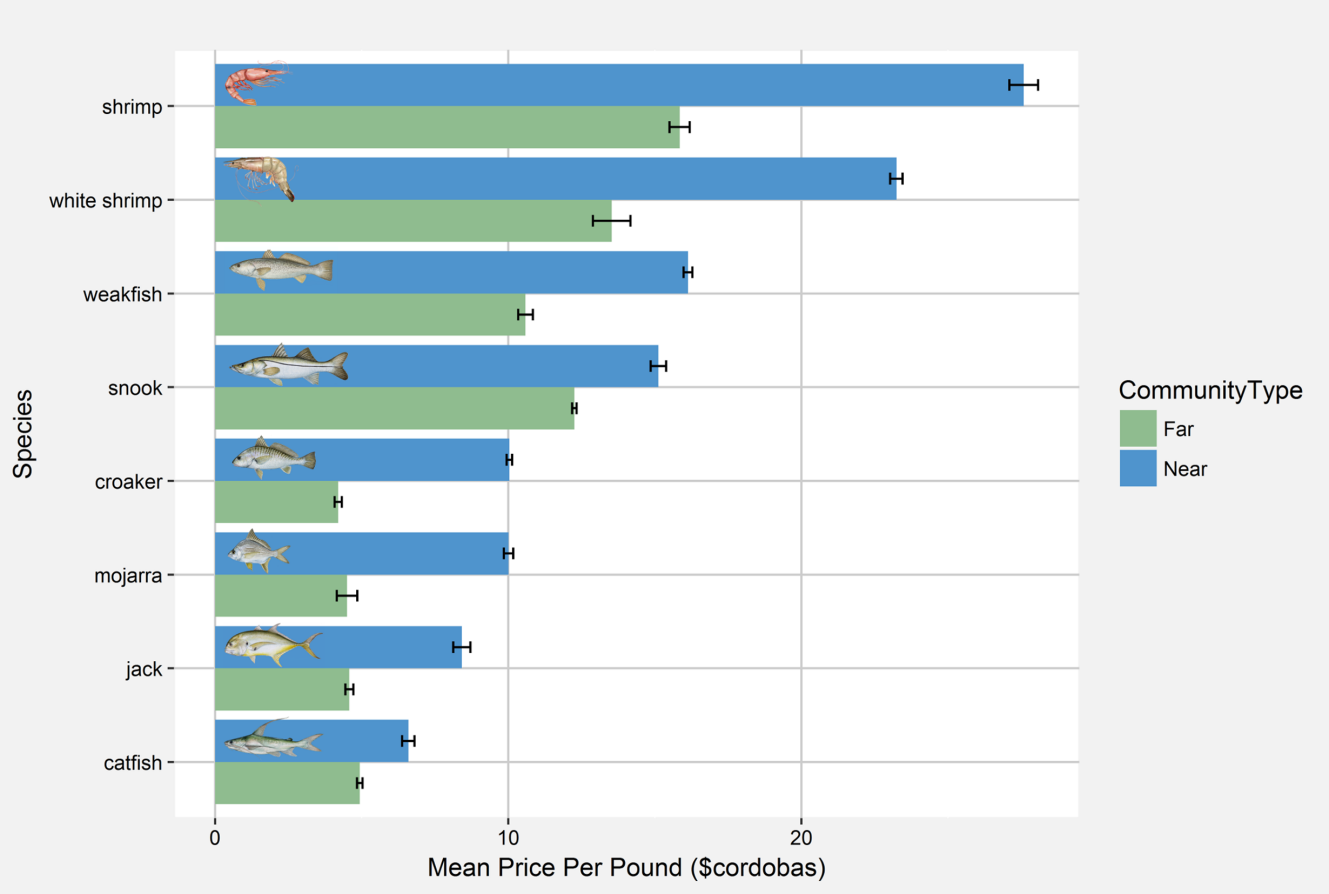
Prices received for commonly caught fish. A comparison of mean price per pound in cordobas between near and far communities for the nine most commonly caught marine species based on total weight between the years 2010 and 2012. Error bars represent the standard error of the mean. Species from top to bottom are *Farfantepenaeus duorarum* (shrimp), *Litopenaeus setiferus* (white shrimp), *Cynoscion spp*. (weakfish), *Centropomus undecimalis* (snook), *Micropogonias spp*. (croaker), *Eugerres plumieri* (mojarra), *Caranx hippos* (jack), and *Bagre marinus* (catfish). The six fish species illustrations are reprinted under a CC BY license with permission from Diane Peebles, 1992, 1992, 1992, 1998, 1992, and 1992 (S1 File). The two shrimp species illustrations are reprinted from Wikimedia Commons and are works in the public domain of the United States (S2 and S3 Files).

**Fig 6:**
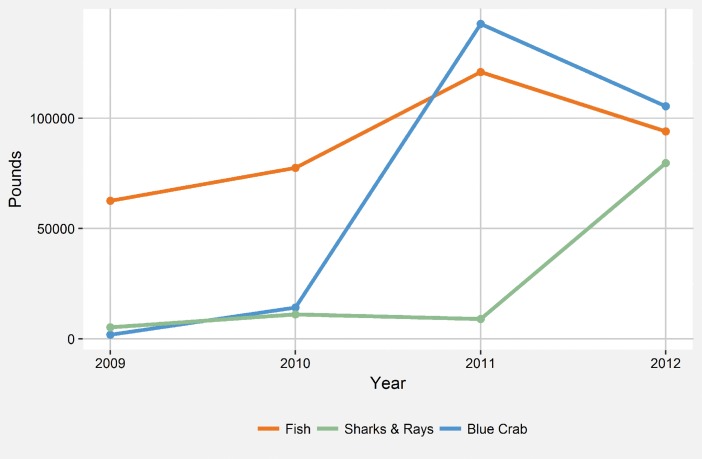
Total landings for fish, blue crab, and sharks and rays between 2009 and 2012. Total landing weight in pounds for fish, blue crab, and sharks and rays for the Pearl Lagoon municipality between 2009 and 2012. Source: Nicaragua INPESCA Fishing Yearbooks 2009, 2010, 2011, and 2012.

**Fig 7:**
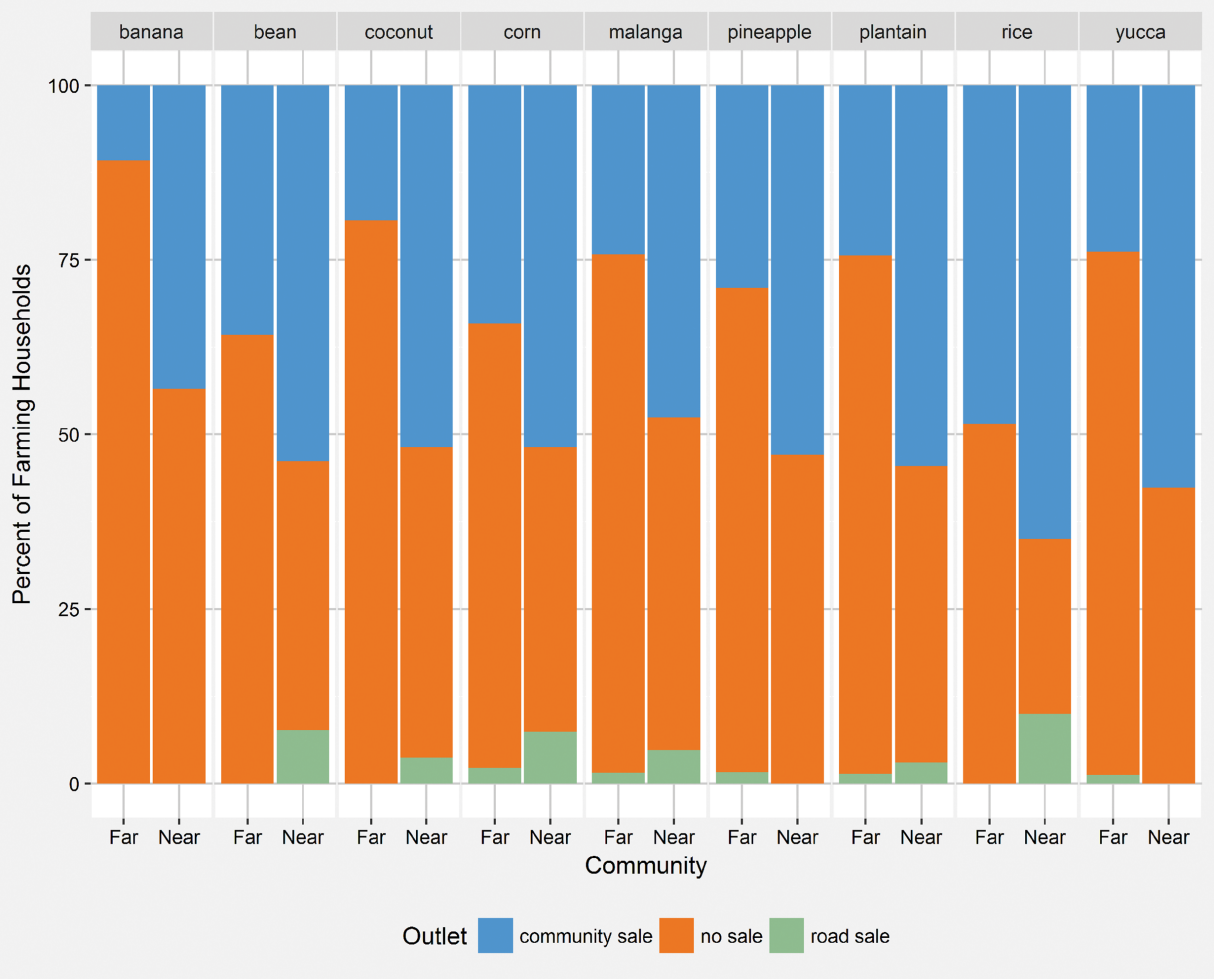
Sales outlets for commonly grown crops for communities near and far from the new road. A comparison of sale outlets for the nine most commonly planted agricultural crops between communities near and far from the new road. Community sales include both sales to small community vendors and other individuals. Road sales are sales to outside vendors in the village of Pearl Lagoon. Households not engaged in commercial exchange may either use crops for subsistence purposes or gifting.

Looking further into the distinctions between communities near and far from the new road, based on nearly 4,000 unique fishing trips, fishers in communities near the new road consistently received higher, often times substantially higher, prices for the eight marine species accounting for 95% of the total catch weight between 2010 and 2012 (Fig 5). Across the lagoon communities, while fish landings increased modestly between 2009 and 2012, landings for blue crab, rays, and sharks increased greatly from roughly 2,000 and 5,000 pounds of blue crab and sharks/rays in 2009 to 105,000 and 80,000 pounds respectively (Fig 6). Considering the commercialization of agriculture, across the lagoon, there were very few households selling any of the nine most commonly planted crops either locally or to road vendors. Commercial sales, although rare, are more common in communities nearer the road. In communities farther from the new road, farming households most commonly (i.e. greater than 50% of households for each crop) do not sell their crops to either community or road vendors (Fig 7).

## Discussion

Farming and fishing exit and entry decisions during a period of rapid globalization showed strong spatial and temporal patterns in Nicaragua's Pearl Lagoon Basin. Households in communities nearer to the new road were less likely to exit fishing–despite a deteriorating fishery throughout the lagoon–as compared to households farther from the road. This result is contrary to prior work finding fishers more willing to exit under hypothetical declines in catch [34], while corroborating more recent findings that fishers, again in response to hypothetical scenarios, were less willing to exit in communities with greater infrastructure and economic activity [36].

Lagoon fisheries have been in a continual state of decline since the early 1990s, reflected by declining biomass, mean trophic level, landings, and catch per unit effort. Over this same period, mean gear size, transport capacity, the number of boat motors, and the number of fish buyers have increased [12]. The observed spatial patterns in livelihood changes appear to be driven by dissimilar access to new markets. At its terminus, the road has generated new opportunities for fishermen near the town of Pearl Lagoon by increasing access to Pacific-based markets and fish buyers. East Asian demand has also driven market development for blue crab [31], sharks and rays (Fig 6). The authors have also witnessed a growing market for jellyfish and sea cucumber. Interestingly, the only distant community with increasing participation in fishing (Fig 3), however slight, was Orinoco, the largest and most developed of the four distant communities. Orinoco has more frequent and rapid boat transportation to the market towns of Pearl Lagoon and Bluefields, and road vendors have established purchase agreements with local fish buyers and the Orinoco fish cooperative (K. Stevens, unpublished data). The marked differences in prices received by fishers in communities nearer and farther from the new road for the most commonly caught species (Fig 5) are further evidence that market access is an important driver of fishers’ livelihood decisions.

Compared to the marine sector, road completion has not generated comparable market opportunities for agriculture [31]. Very few farming households sell their products to road vendors, and a majority sell none of the nine most commonly grown crops (Fig 7). In addition, many agricultural products sold by vendors from the Pacific and Highland regions of Nicaragua are being imported more cheaply. Thus, local farmers may face greater competition, particularly in communities nearer the road. This may explain why farmers in distant communities were less likely to exit. But, as local farmers primarily grow for their own consumption, other reasons might explain spatially-driven, farmer exit decisions beyond greater competition.

A greater reluctance to exit farming in distant communities may be tied to food security. Our results provide modest support for this conclusion. First, farming in distant communities may act as a food security safety net while declining marine resources become increasingly difficult to capitalize farther from the road [43]. This is evidenced by our finding that farmers with greater food self-sufficiency in 2010 were less likely to exit in 2012. Households in communities nearer the road rely more heavily on purchased food, disincentivizing farming. Finally, increasing immigration by Mestizos may incentivize active use of farm land in distant communities in order to secure land susceptible to the advancing agricultural frontier amid weak property rights and poor title enforcement (K. Stevens, personal communication).

In addition to spatially-driven variation in market dynamics at the community-level, household-level characteristics also affected coastal livelihood transitions. An increase in household wealth was the most important predictor of entering fishing. Similarly, larger households were associated with greater odds of entering fishing, which requires both economic and labor investments. While local farmers can freely access communal lands and engage in small-scale, non-mechanized agriculture with few inputs [43], fishers have greater capital costs in gear and fuel. Households with greater assets may be freer to engage in riskier endeavors, like investment in an increasingly commercialized yet declining fishery.

Crop diversity was associated with smaller odds of exiting farming. Crop diversity may bolster household resilience to dynamic markets and natural resource fluctuations [44], thus incentivizing households to remain in farming despite the risks associated with increasing ties to external markets. While the parameter estimate for the effect of catch diversity on fishing exit was also negative, there was little support for this relationship (RI = 0.50 and 95% CI = -0.72 to 0.11). While the livelihoods of fishing and farming showed clear spatial patterns among near and distant communities, there was little interaction between these livelihoods at the household level. Engagement in forest product collection, however, did correlate with household farming and fishing decisions. Households participating in forest product gathering were more likely to enter farming and less likely to exit both farming and fishing. Because farm land is interspersed with secondary forest, it is convenient for farmers to gather forest products. For fishers, on the other hand, forest product gathering may diminish the risks associated with increasingly commercialized yet declining fisheries.

Our results suggest that increased market access and related price differentials for natural resource products are a critical driver of changing livelihoods on the Caribbean coast of Nicaragua. However, other explanations should be considered. Land availability differs somewhat between communities nearer and farther from the new road. In nearer communities, new farming entrants are likely to have to travel farther to access their plots, but land is available. At the same time, however, communities farther from the road also face constraints to accessing new plots due to Mestizo colonization. Additionally, while our analyses attempted to control for differences due to ethnicity, this was based on language, rendering us unable to distinguish between Garifuna and Creole people. The northern communities of Orinoco, Brown Bank, and La Fe all have large Garifuna communities whereas Pearl Lagoon, Awas, and Raitipura have large Creole and Miskitu populations. However, Kakabila, also a northern community, has a significant Miskitu population, and thus the extent to which ethnicity tracks with our distinction between near and far communities is good but not perfect. Also, as Williams [32] notes, ethnicity is often a “fluid, socio-political identity that shapes and is shaped by opportunities and constraints.” Still, we acknowledge the possibility that ethnicity plays a larger role in explaining livelihood decisions than we are able to explain. Similarly, our categorization of communities near and distant to the new road precludes an analysis of characteristics unique to each community that may drive exit and entry decisions. For example, among distant communities, wealth may demonstrate a different relationship with household livelihood decisions. The small populations of many of our study communities along with small samples after dividing our original sample by seven communities, by farming and fishing households, and by livelihood decision (i.e. exit and entry), makes intra-community analysis difficult. Finally, as noted above, communities near and distant to the new road differ in many ways in addition to market access including wealth, farm size, food self-sufficiency, and livelihood diversity (Fig 4), and some of these differences may have existed prior to the completion of the new road. The larger town of Bluefields, for example, south of our study communities, is accessible by boat from each of our study communities and was so prior to the road. Undoubtedly, however, over the study period, a major driving force affecting and differentiating our study communities, based on several years of personal communications with area residents and observations by the authors, is the road.

## Conclusion

We found Costeño decisions on whether to enter or exit farming and fishing demonstrated clear temporal and spatial patterns, likely caused by different access to new market opportunities via a recently completed road on Nicaragua's Caribbean Coast. Fishers in communities nearer the road are doubling down on a declining fishery, seemingly reluctant to exit due to higher prices and new market opportunities. Fishing households in communities farther from the new road and its market access, are more likely to exit. Farmer exit decisions follow an opposite pattern. Those in near communities are transitioning away from farming, facing greater market competition via the road, while farmers in distant communities continue to rely on agriculture, perhaps embracing the food security that subsistence farming affords. The natural resource implications of these livelihood decisions are potentially profound as they provide novel linkages between terrestrial and marine ecosystems.

Across the entire region, there were tradeoffs between fishing and farming while within households (Fig 3), there were tradeoffs between forest product gathering and the two primary livelihoods of fishing and farming. The new transnational road may be incentivizing livelihood adaptations across these three natural resource-based livelihoods spanning both terrestrial and marine ecosystems to as of yet unknown effect. With fewer market opportunities for marine products in communities farther from the road, fishers may rely more on terrestrial-based livelihoods, putting greater pressure on local forests, fauna, soils, and water ways. For example, while forest product gathering has historically been subsistence orientated on the Caribbean coast, the new road has spurred larger-scaled commercial exploitation including that for high-value timber, culminating in recently announced export controls for rosewood by the Convention for the International Trade of Endangered Species [45]. Farming and forestry can in turn create negative feedbacks for marine systems due to greater sedimentation, runoff, and degradation of mangroves and marine resources. Caribbean coast forests, part of the Meso-American Biological Corridor, are home to several charismatic mammal species including the IUCN classified endangered Baird’s tapir (*Tapirus bairdii*) [46]. A recent analysis of jaguar (*Pathera onca*) habitat in Central and South America found that among Central American countries, Nicaragua saw the largest percentage, nearly 11 percent, decrease in forest change in unprotected areas [47].

Greater competition in farming and more market opportunities in fishing, particularly in communities nearer the new road, may be spurring the commercialization of other marine resources. [48], for example, report increased commodification of the green turtle (*Chelonia mydas*) fishery beyond its traditional, cultural importance. Since the completion of the new road, green turtles are being trucked inland to Mestizo communities with no historical custom of consuming turtle meat.

Future research identifying these trans-ecosystem tradeoffs in rapidly changing coastal systems is crucial to mitigating the ill-effects of infrastructure development. Our findings support the need to undertake a trans-ecosystem approach to natural resource management and development policy, particularly in coastal regions experiencing rapid globalization. While evaluating these linkages is difficult, mitigating their negative effects is made more so because of institutional fragmentation and isolation between natural resource governance agencies that are often tasked with the exclusive management of either terrestrial, marine, or freshwater ecosystems, common in Nicaragua and elsewhere despite the strong socio-economic and ecological linkages between the three, particularly in coastal regions.

Beyond external changes, because management often focusses on one sector and its related livelihood, policy interventions may have unintended consequences for livelihood-linked ecosystems. For example, restricting marine harvest may increase terrestrial-based hunting, land clearing, and cattle ranching. Similarly, changing land tenure rules, often done in conjunction with infrastructure development [49], may add pressure to marine systems. Moreover, even when natural resource agencies adopt a trans-ecosystem approach, the linkages, ecological or socio-economic, at the land-sea interface are sometimes spatially explicit as shown here. Different management plans are likely required for the northern and southern ends of Pearl Lagoon because of the very different effects of market access on the primary natural resource-based livelihoods.

It has long been recognized that livelihoods are connected because households practice multiple livelihoods, which they periodically rebalance in the face of changing external forces. Our study reveals spatially explicit and intra-household linkages between livelihood transitions in response to new infrastructure and market access. We urge the science and policy communities to increase their attention to linked livelihoods and their scrutiny of the trans-ecosystem implications of linked livelihood transitions for coastal, social-ecological systems.

## Supporting information

S1 File Permissions for fish images in Fig 5. Permission granted from Diane Peebles for *Cynoscion spp*. (weakfish), *Centropomus undecimalis* (snook), *Micropogonias spp*. (croaker), *Eugerres plumieri* (mojarra), *Caranx hippos* (jack), and *Bagre marinus* (catfish) in Fig 5.
(PDF)

S2 File Public domain image of shrimp in Fig 5. The image of *Farfantepenaeus duorarum* (shrimp) in Fig 5 is in the public domain of the United States.
(PDF)

S3 File Public domain image of white shrimp in Fig 5. The image of *Litopenaeus setiferus* (white shrimp) in Fig 5 is in the public domain of the United States.
(PDF)
