# Supplementary material for: Coastal livelihood transitions under globalization with implications for trans-ecosystem interactions
Source: PLoS One. 2017 Oct 27;12(10):e0186683. doi: 10.1371/journal.pone.0186683 (PMC5659644; doi:10.1371/journal.pone.0186683)
Supplement: S1 File — Permission granted from Diane Peebles for Cynoscion spp. (weakfish), Centropomus undecimalis (snook), Micropogonias spp. (croaker), Eugerres plumieri (mojarra), Caranx hippos (jack), and Bagre marinus (catfish) in Fig 5. (PDF) [file pone.0186683.s001.pdf]

From: **Peebles** epeebles@verizon.net
Subject: RE: Fish drawings
Date: August 16, 2017 at 9:10 AM
To: Kramer, Daniel dbk@msu.edu

Daniel,

I will send over the images via my Hightail account shortly. With regard to the croaker name, I can't allow it to be mislabeled as a different species (make me look bad as an illustrator), but I suppose it would be okay to call it *Micropogonias spp*. Can you tell me the approximate reproduction size of the images so I know what size files to send to you?

Diane Rome Peebles Fine Art, LLC
P. O. Box 12855
St. Petersburg, FL 33713
(727) 321-5951

www.dianepeebles.com

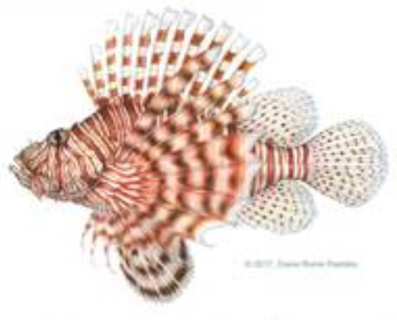

From: Kramer, Daniel [mailto:dbk@msu.edu]
Sent: Friday, August 11, 2017 9:52 AM
To: Peebles
Subject: Re: Fish drawings

Dear Diane,

Thanks so much. Below are the illustrations we'd like to use. I am not sure how this works. Do you send me them without the watermark?

The Atlantic croaker is most similar to the species we encounter in Nicaragua, the Whitemouth Croaker (Micropogonias furnieri). Would you mind if in our paper we label your image of the Atlantic Croaker either Whitemouth Croaker (Micropogonias furnieri) or Micropogonias spp.?

By the way, your illustrations are really beautiful.

Dan

Gafftopsail Catfish (Bagre marinus)

Atlantic Croaker (Micropogonias undulatus)

[illegible]

Mojarras (Eugerres plumieri)

Weakfish (Cynoscion regalis)

Common Snook (Centropomus undecimalis)

Crevalle Jack (Caranx hippos)

Daniel Boyd Kramer
Michigan State University
842 Chestnut Road | 365 North Case Hall | East Lansing, MI 48824
517.432.2199 | dbk@msu.edu | www.danielboydkramer.com
James Madison College
Department of Fisheries & Wildlife | College of Agriculture and Natural Reso
MICHIGAN STATE UNIVERSITY

On Aug 11, 2017, at 6:10 AM, Peebles <epeebles@verizon.net> wrote:

Dan,

Yes, that is fine to use the fish illustrations with the standard licensing fees waived for the purpose you described. Where appropriate, please include the credit information, © Diane Rome Peebles.

Diane Rome Peebles Fine Art, LLC
P. O. Box 12855
St. Petersburg, FL 33713
(727) 321-5951

www.dianepeebles.com
<image001.jpg>

From: Kramer, Daniel [mailto:dbk@msu.edu]
Sent: Thursday, August 10, 2017 3:48 PM
To: epeebles@verizon.net
Subject: Fish drawings

Dear Ms. Peebles,

I am writing to request permission to use some of your images in an academic manuscript. I have attached the proposed figure in which I plan to use the images so you understand their use.

Below is the request that the journal advises I send to you.

"I request permission for the open access journal PLOS ONE to publish XXX

I request permission for the open-access journal PLOS ONE to publish XXX under the Creative Commons Attribution License (CCAL) CC BY 4.0 (http://creativecommons.org/licenses/by/4.0/). Please be aware that this license allows unrestricted use and distribution, even commercially, by third parties. Please reply and provide explicit written permission to publish XXX under a CC BY license."

I am interested in the following: mojarra, weakfish, snook, catfish, black drum (croaker), jack, white shrimp, and red shrimp. I am not sure whether you have images of all of these but I know there are at least two that you do (mojarra and black drum).

If you agree in principle, I can look at your web site and find the images I would like to use.

Sincerely,

Dan

<image002.png>

Daniel Boyd Kramer
Michigan State University
842 Chestnut Road | 365 North Case Hall | East Lansing, MI 48824
517.432.2199 | dbk@msu.edu | www.danielboydkramer.com
<image003.jpg>

Virus-free. www.avg.com
