# Supplementary material for: Coastal livelihood transitions under globalization with implications for trans-ecosystem interactions
Source: PLoS One. 2017 Oct 27;12(10):e0186683. doi: 10.1371/journal.pone.0186683 (PMC5659644; doi:10.1371/journal.pone.0186683)
Supplement: S3 File — The image of Litopenaeus setiferus (white shrimp) in Fig 5 is in the public domain of the United States. (PDF) [file pone.0186683.s003.pdf]

File:Litopenaeus setiferus.png

From Wikimedia Commons, the free media repository

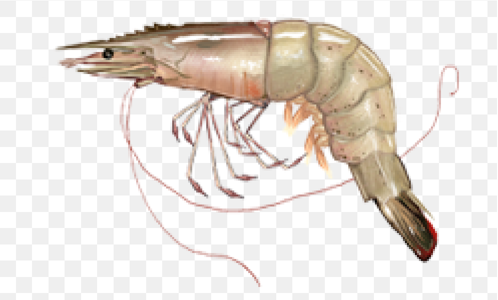

No higher resolution available.

Litopenaeus_setiferus.png (248 × 150 pixels, file size: 46 KB, MIME type: image/png)

Open in Media Viewer

Summary

**Description**	**English:** White shrimp, *Litopenaeus setiferus*
**Date**	4 November 2012, 18:31:11
**Source**	NOAA FishWatch (http://www.fishwatch.gov/seafood_profiles/species/shrimp/species_pages/white_shrimp.htm)
**Author**	Unknown

Licensing

*This work is in the **public domain** in the United States because it is a work prepared by an officer or employee of the United States Government as part of that person's official duties under the terms of* Title 17, Chapter 1, Section 105 of the US Code. *See Copyright.*

<u>Note</u>: This only applies to original works of the Federal Government and not to the work of any individual U.S. state, territory, commonwealth, county, municipality, or any other subdivision. This template also does not apply to postage stamp designs published by the United States Postal Service since 1978. (See § 313.6(C)(1) (http://copyright.gov/comp3/chap300/ch300-copyrightable-authorship.pdf) of Compendium of U.S. Copyright Office Practices). It also does not apply to certain US coins; see The US Mint Terms of Use (http://www.usmint.gov/policy/?action=TermsOfUse).

This file has been identified as being free of known restrictions under copyright law, including all related and neighboring rights. (https://creativecommons.org/publicdomain/mark/1.0/deed.en)

File history

Click on a date/time to view the file as it appeared at that time.

	Date/Time	Thumbnail	Dimensions	User	Comment
current	**05:34, 4 November 2012**	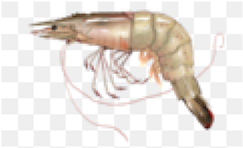	248 × 150 (46 KB)	Epipelagic (talk | contribs)	User created page with UploadWizard

- You cannot overwrite this file.

File usage on Commons

There are no pages that link to this file.

File usage on other wikis

The following other wikis use this file:

- Usage on ca.wikipedia.org
  - Litopenaeus setiferus
- Usage on ceb.wikipedia.org
  - Litopenaeus setiferus
- Usage on en.wikipedia.org
  - Litopenaeus setiferus
- Usage on fa.wikipedia.org
  - میگوی خاکستری
- Usage on fi.wikipedia.org
  - Dendrobranchiata
- Usage on fr.wikipedia.org
  - Penaeidae
- Usage on no.wikipedia.org
  - Litopenaeus setiferus
- Usage on pt.wikipedia.org
  - Camarão-lixo
- Usage on sv.wikipedia.org
  - Litopenaeus
  - Litopenaeus setiferus
- Usage on war.wikipedia.org
  - Litopenaeus setiferus
- Usage on www.wikidata.org
  - Q5221424
  - User:Achim Raschka/Erstbeschreibungen Linnaeus

Metadata

This file contains additional information such as Exif metadata which may have been added by the digital camera, scanner, or software program used to create or digitize it. If the file has been modified from its original state, some details such as the timestamp may not fully reflect those of the original file. The timestamp is only as accurate as the clock in the camera, and it may be completely wrong.

Retrieved from "https://commons.wikimedia.org/w/index.php?title=File:Litopenaeus_setiferus.png&oldid=218448020"

- This page was last edited on 24 November 2016, at 02:55.
- Text is available under the Creative Commons Attribution-ShareAlike License; additional terms may apply. By using this site, you agree to the Terms of Use and Privacy Policy.
